# Cationic and Biocompatible Polymer/Lipid Nanoparticles as Immunoadjuvants

**DOI:** 10.3390/pharmaceutics13111859

**Published:** 2021-11-04

**Authors:** Yunys Pérez-Betancourt, Péricles Marques Araujo, Bianca de Carvalho Lins Fernandes Távora, Daniele Rodrigues Pereira, Eliana Lima Faquim-Mauro, Ana Maria Carmona-Ribeiro

**Affiliations:** 1 Biocolloids Laboratory, Departamento de Bioquímica, Instituto de Química, Universidade de São Paulo, Avenida Professor Lineu Prestes, 748 Butantan, São Paulo 05508-000, Brazil; y.betancourt@usp.br (Y.P.-B.); periclesaraujo@usp.br (P.M.A.); 2 Immunopathology Laboratory, Butantan Institute, Avenida Vital Brasil, 1500 Butantan, São Paulo 05503-900, Brazil; bianca.tavora@butantan.gov.br (B.d.C.L.F.T.); rodriguesdaniele663@gmail.com (D.R.P.); eliana.faquim@butantan.gov.br (E.L.F.-M.); 3 Departamento de Imunologia, Instituto de Ciências Biomédicas, Universidade de São Paulo, Avenida Professor Lineu Prestes, 1730 Butantan, São Paulo 05508-000, Brazil

**Keywords:** biocompatible polymer, cationic lipid, poly(methyl methacrylate), dioctadecyl dimethyl ammonium bromide, hybrid nanoparticles, ovalbumin, dynamic light scattering, nanoparticles cytotoxicity, delayed type hypersensitivity, cytokines profile

## Abstract

Nanostructures have been of paramount importance for developing immunoadjuvants. They must be cationic and non-cytotoxic, easily assembling with usually oppositely charged antigens such as proteins, haptens or nucleic acids for use in vaccines. We obtained optimal hybrid nanoparticles (NPs) from the biocompatible polymer poly(methyl methacrylate) (PMMA) and the cationic lipid dioctadecyl dimethyl ammonium bromide (DODAB) by emulsion polymerization of methyl methacrylate (MMA) in the presence of DODAB. NPs adsorbed ovalbumin (OVA) as a model antigen and we determined their adjuvant properties. Interestingly, they elicited high double immune responses of the cellular and humoral types overcoming the poor biocompatibility of DODAB-based adjuvants of the bilayer type. The results suggested that the novel adjuvant would be possibly of use in a variety of vaccines.

## 1. Introduction

In vaccine design, cationic nanostructures are very important as adjuvants for carrying antigens. Their nanometric size avoids retention by physiological barriers at the site of injection allowing them to reach lymph nodes and dendritic cells more easily [1,2,3,4,5,6]. They can be obtained from a variety of lipids, polymers or lipids and polymers, mostly as bilayer vesicles [7,8], open bilayer disks [9,10,11,12,13], lipid nanoparticles [14] or hybrid nanoparticles [15,16,17,18,19,20,21].

In particular, the cationic and synthetic lipid dioctadecyl dimethyl ammonium bromide (DODAB) self-assembles both as closed and large bilayer vesicles [8,22] or as open and cationic bilayer fragments or nanodisks (DODAB BF) with sizes below 100 nm that fuse upon relaxation of the surface potential due to the addition of monovalent salt [23]. DODAB bilayers can combine with a broad variety of antigens or enhancers of the immune response, such as proteins [7,10,24,25], peptides [18,26,27], oligonucleotides [11,28], mononucleotides [29,30] or nucleic acids [31,32], driving an enhanced cell-mediated immune response, but a poor humoral response in several instances [7,10,11,15].

The versatile character of the DODAB molecules and their assemblies directed our research to characterize the many DODAB interactions with oppositely charged particles such as polystyrene sulfate latexes [15,18,33,34,35] or silica [36,37,38,39]. This basic knowledge led to the optimized deposition of supported DODAB bilayers on polystyrene sulfate (PSS) nanoparticles [15,18,35] or silica [38,39] as suitable immunoadjuvants and antigen carriers. The preparation and characterization of PSS/DODAB NPs, where a single DODAB bilayer surrounds each PSS NP was described [18].

The good miscibility of DODAB with the biocompatible polymer poly(methylmethacrylate) (PMMA) [16,40] motivated the preparation of hybrid PMMA/DODAB NPs by emulsion polymerization of methylmethacrylate (MMA) in the presence of DODAB. These hybrid, cationic and nanometric PMMA/DODAB NPs were described previously [19]. Here we test the application of these PMMA/DODAB NPs as immuno-adjuvants carrying ovalbumin (OVA) as a model antigen. The advantages of the PMMA/DODAB NPs were biocompatibility, high affinity for oppositely charged antigens and induction of a mixed Th2/Th1 immune response.

## 2. Materials and Methods

### 2.1. Materials

MMA, azobisisobutyronitrile (AIBN), NaCl, DODAB, chloroform, grade V ovalbumin, ortho-phenilenediamine dihydrochloride (OPD), peroxidase—labeled streptavidin, and concanavalin A—were purchased from Sigma-Aldrich (Saint Louis, MO, USA). OVA stock solution was prepared in Milli-Q water at 15 mg/mL and was purified by chromatography (polymyxin B columns; Pierce Biotechnology, Rockford, IL, USA), analyzed by the bicinchoninic acid (BCA) assay, and adjusted to 10 mg/mL. Al(OH)_3_ as adjuvant was purchased from Sanofi—Synthelabo, RJ, Brazil. Cellulose acetate membranes (12,400 g/mol molecular weight cut off) were used for dialyzing the nanoparticles dispersions. Analytical grade reagents were used throughout without purification.

**Scheme 1:**
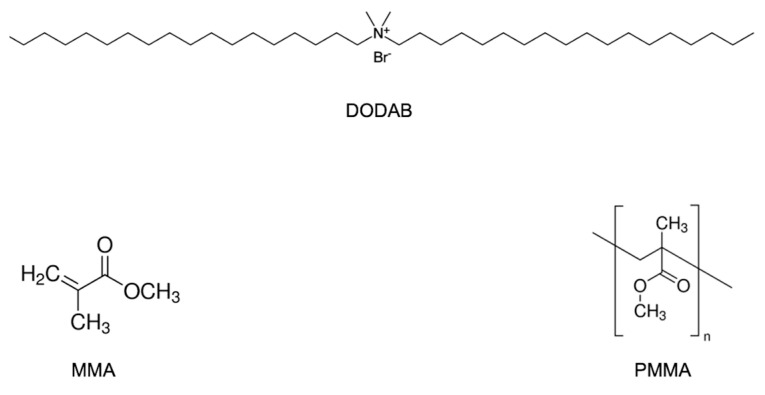
Chemical structures of dimethyl dioctadecyl ammonium bromide (DODAB), methyl methacrylate (MMA) and polymethyl methacrylate (PMMA).

The chemical structures for MMA, DODAB and PMMA are presented in Scheme 1.

### 2.2. Spreading of DODAB Films, Preparation of DODAB Bilayer Fragments (DODAB BF) and Synthesis of PMMA/DODAB NPs

Two different DODAB dispersions in water solution were prepared: (1) dispersions from DODAB films [27,41]; (2) DODAB bilayer fragments (BF) [8,9,42]. DODAB films on the bottom of glass tubes were obtained from a 10 mM DODAB solution in chloroform submitted to evaporation under nitrogen flow. For preparing DODAB BF, DODAB powder was sonicated for 15 min in 1 mM NaCl water solution using a macroprobe (85 W) and then centrifuged for pelleting titanium ejected from the tip (9300× *g*/1 h/4 °C).

The synthesis of PMMA/DODAB NPs was carried out by polymerizing MMA (emulsion polymerization) in the presence of DODAB and AIBN initiator [16,19,43]. In the tubes containing the dry DODAB films or DODAB bilayer fragments (BF), MMA (0.1 to 0.56 M), 0.036 g AIBN and 1 mM NaCl solution were added to a final volume of 10 mL and placed in a water bath at 70–80 °C for 1 h with periodic vortexing before exhaustive dialysis for eliminating unreacted monomer (4 L water, 6 times change, 72 h/Milli-Q water).

### 2.3. Determining Physical Properties of NPs by Dynamic Light-Scattering (DLS)

Dispersions were diluted 20 times for zeta-average diameter (Dz), zeta-potential (ζ) and polydispersity (P) DLS measurements using a Zeta-Plus Zeta-Potential Analyzer (Brookhaven Instruments Corporation, Holtsville, NY, USA), which was equipped with a 677 nm laser and dynamic light-scattering (DLS) at 90° for particle sizing. From fluctuations of scattered light intensity, the decay times of the fluctuations can be related to the diffusion constants and, therefore, to the sizes of the particles. Small particles moving rapidly cause faster decaying fluctuations than large particles moving slowly. The decay times of these fluctuations are determined in the time domain using a correlator. The fluctuating signal is processed by forming the autocorrelation function which decays exponentially with time so that the relaxation of the fluctuations is directly related to the decay constant (Γ). The decay constant is given by Equation (1):

Γ = Dq^2^ (1)

where q depends on the scattering angle, the wavelength of the laser light and refractive index of the suspending liquid and D is the translational diffusion coefficient.

Hydrodynamic particle diameter (Dz) is inversely related to D by the Stokes-Einstein Equation (2):

Dz = kT/(3πηD)(2)

where k is the Boltzmann’s constant, T is temperature in Kelvin, η is the viscosity of the suspending liquid, and D is the diffusion coefficient. Mean diameters were obtained by fitting data to log-normal size distributions that do not discriminate between one, two, or more different populations and always consider all scattering particles as belonging to one single Gaussian population. For the size distribution data, fitting was performed by the apparatus software using the non-negatively constrained least-squares (NNLS) algorithm, which is a model-independent technique allowing multimodal distributions to be achieved [44].

In order to define a relative width of size distributions, the polydispersity P is given by Equation (3):

P = µ_2_Γ^2^ (3)

where μ_2_ is proportional to the variance of the “intensity” weighted diffusion coefficient distribution and carries information on the width of the size distribution. Polydispersity (P) has no units. It is close to zero (0.000 to 0.020) for monodisperse or nearly monodisperse samples, small (0.020 to 0.080) for narrow distributions and larger for broader distributions. ζ was determined from electrophoretic mobility (µ) in 1 mM NaCl and the Smoluchowski equation (Equation (4)):

ζ = μη/ε(4)

where η is the medium viscosity and ε the medium dielectric constant. DODAB differs from surfactants by being a lipid.

### 2.4. Determination of Solid Content and DODAB Concentration in NPs

Solid contents (mg/mL) in the dispersions were quantified by weighing 1 mL NPs dispersions before and after lyophilization as previously described [16,17,19,45]. Centrifuging dispersions (1 h/10,000 rpm) and pelleting NPs allowed the determination of DODAB concentration in the supernatant from halide microtitration [46]. DODAB concentration in NPs was obtained by subtracting DODAB concentration in the supernatant from DODAB concentration used for NPs synthesis [21,45].

### 2.5. Determination of Interaction of PMMA/DODAB NPs (20 mg/mL PMMA and 1.6 mM DODAB) and OVA

After washing out DODAB in the supernatant of a PMMA/DODAB NPs dispersion by centrifuging and re-suspending the initial dispersion three times in pure water, an aliquot containing 0.50 mg/mL PMMA and 0.08 mM DODAB interacted with OVA over a range of OVA concentrations during 1 h interaction time. Thereafter, photos, DLS measurements and determination of OVA adsorption onto NPs from OVA analysis in the supernatants of the NPs/OVA mixtures were obtained [47]. Adsorbed [OVA] was obtained after centrifuging (10,000 rpm/1 h/25 °C) by subtracting OVA concentration in the supernatant from the OVA concentration added.

### 2.6. Determination of Cell Viability in the Presence of PMMA/DODAB NPs as Compared to the One with DODAB Vesicles and Bilayer Fragments

Three DODAB adjuvants were tested against cells in culture: PMMA/DODAB NPs, DODAB bilayer vesicles (DODAB LV) and DODAB BF. Stock dispersions of the three adjuvants were: (1) PMMA/DODAB NPs at 20 mg/mL PMMA and 1.6 mM DODAB; (2) DODAB LV at 10 mM DODAB in DODAB dispersions obtained by vortexing the DODAB powder in 1 mM NaCl solution above 47 °C [41]; (3) DODAB BF at 10 mM DODAB obtained by sonication in 1 mM NaCl with a macro-probe. Aliquots of each stock dispersion of adjuvants were mixed with cells’ culture media over a range of DODAB concentrations (0.04, 0.08, 0.12, 0.16 mM DODAB for each adjuvant type). Thereafter 0.1 mL of each adjuvant type and concentration in culture medium were added to subconfluent layers of L929 fibroblasts or J774A.1 macrophages (American Type Culture Collection).

Cell lines were cultured according to standard protocols (90% humidity, 5% CO_2_, 37 °C), RPMI—1640, 5% fetal bovine serum (FBS), 1% penicillin—streptomycin, 2 mM L—glutamine, and 0.25% 2—mercaptoethanol. In a humidified CO_2_ incubator, 0.1 mL of DODAB-containing adjuvant previously diluted in the cells medium (0.1 mL) interacted with plated cells (10,000 cells/well) for 3 and 24 h. The 3-(4,5-dimethylthiazol-2-yl)-2,5-diphenyl tetrazolium bromide (MTT) assay evaluated cytotoxicity. Ten microliters of MTT solution in PBS (5 mg/mL MTT) was added per well. This stock MTT solution was previously filtered for sterilization and removal of insoluble residues. After incubation (37 °C/2 h), supernatant withdrawing, and mixing of adhered cells with 100 µL isopropanol solution in 0.04 N HCl, the absorbance at 570 nm was determined on an ELISA reader. One hundred percent of cell viability was given by cells in the culture medium only.

### 2.7. Immunization Protocol

Animals were immunized subcutaneously according to four groups with five BALB/c male mice each. They were injected with 0.1 mL of solution or dispersion on the base of the tail at two separate sites with 0.1 mL each (0.2 mL per animal in total). The composition of solutions or dispersions used for immunization is in Table 1. Animals received a booster with the same priming dose on day 21 after immunization.

### 2.8. OVA-Specific IgG1 and IgG2a Production by ELISA in Immunized Mice

Animals’ blood was collected from the ophthalmic plexus at days 14, 21 and 28 postimmunization for serum individual analysis by indirect enzyme-linked immunosorbent assay (ELISA). Microtiter plates (Costar Corning Inc., Kennebunk, ME, USA) were incubated overnight at 4 °C with 0.1 mL OVA solution at 0.010 mg/mL in 0.01 M PBS, pH 7.2. Gelatin 3% in PBS blocked the wells for 2 h before adding serially diluted serum and further incubating the microplates for 1 h at 37 °C. 0.100 mL of goat anti-mouse biotin-conjugated IgG1 (1:1000) or IgG2a (1:500) (Southern Biotechnology Associates, Birmingham, AL, USA) were added per well for incubation (1 h/37 °C). Thereafter, peroxidase-labeled streptavidin (0.100 mL/diluted 1:3000) was added and incubated for 1 h at 37 °C. Three-times washing was performed after each incubation step with PBS containing 0.05% Tween 20 (PBST). An amount of 0.1 mL of ortho-phenilenediamine dihydrochloride (OPD) substrate solution at 1 mg/mL and H_2_O_2_ (1 μL/mL) in 0.1 M citrate-phosphate at pH 5.0) were added per well for 15 min incubation at 25 °C. An amount of 0.050 mL of a H_2_SO_4_ solution (2 M) terminated the reaction. Optical density at 492 nm was read in an ELISA apparatus (Multiskan Ex, Thermo Electron Corporation, Waltham, MA, USA). Samples were diluted to fit inside the 0–1 absorbance range and data were shown. The results were expressed for diluted serum samples as the mean values ± standard deviation. For IgG1 dilution of 1:1024 corresponded to the primary response and 1/512,000, to the secondary one. For IgG2a, primary and secondary responses were present at 1:32 and 1:512 dilutions.

### 2.9. Determination of Delayed-Type Hypersensitivity Reaction (DTH)

DTH associated with cell-mediated immune response was evaluated from footpad swelling in mice [7,11,38,48]. At day 5 postimmunization, mice received injections of denaturated OVA in saline solution (0.030 mL of a 2 mg/mL OVA) at the left-hind footpad. Negative control was 0.030 mL of saline injected at the right-hind footpad of each animal. Footpad swelling at 24 h after injection was measured with a Mitutoyo digital micrometer. The difference between swelling of left and right hind footpads of a given 24 h after injection was taken as the individual footpad swelling. The mean footpad swelling ± standard deviation could be calculated considering all five animals in each group.

### 2.10. Determination of Cytokines Profile from Cultured Spleen Cells

Two weeks after the booster, spleen cells from spleens of five immunized mice of the same group were cultured in RPMI 1640 medium with 2 mM L—glutamine, 5% FBS and 0.25% 2-mercaptoethanol [21,49]. Suspensions (8 × 10^6^ cells/mL) distributed in tissue culture plates were incubated with medium containing 250 μg/mL OVA in a humidified CO_2_ incubator for 72 h. Negative and positive controls were medium only or 2.5 μg/mL concanavalin A (Con-A), respectively. Thereafter, plates were centrifuged (5 min/1500 rpm) and the supernatants collected for determining IL-2, IL-4, IL-10 and IFN-γ (sandwich ELISA kit, catalog number #88-7711-44, Thermo Fisher Scientific, Waltham, MA, USA). Detection limits were 30 (IL-10), 2 (IL-2), 4 (IL-4) and 15 pg/mL (IFN-γ); cytokine concentrations were expressed as mean of duplicate assays ± mean standard deviation.

### 2.11. Statistics Tests

The significance of results for different groups was evaluated using a two-way ANOVA variance analysis and the multiple comparisons Tukey’s test. *p* < 0.05 meant significant differences between results. The software for statistical analysis was provided by the Origin 2018 program available from the Origin Lab Corporation, Northampton, MA, USA.

## 3. Results and Discussion

### 3.1. Synthesis of Nanoparticles (NP), NPs Physical Properties and Cytotoxicity against Cultured Cells

**Figure 1:**
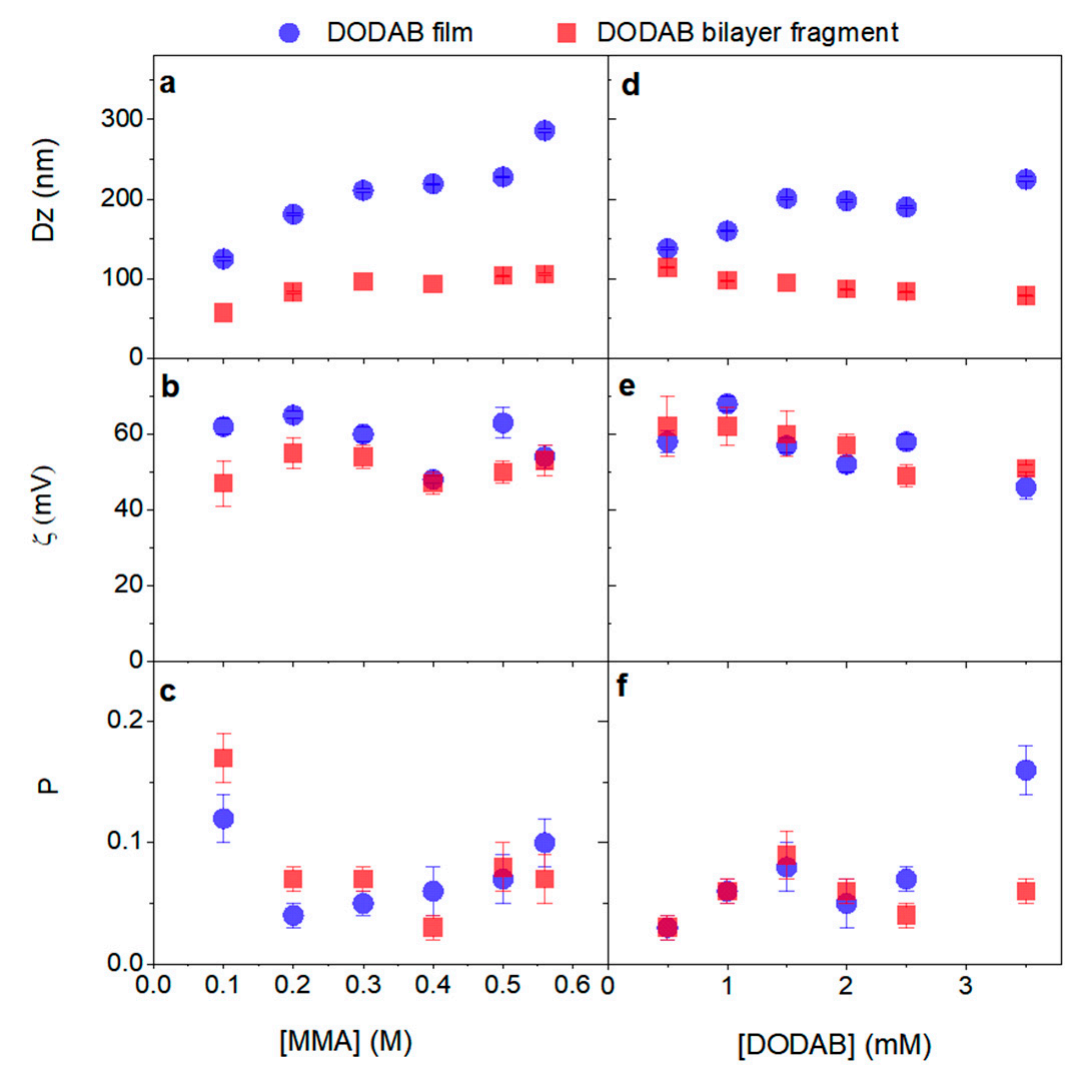
Effect of methyl methacrylate (MMA) concentration (**a**–**c**) or dioctadecyl dimethyl ammonium bromide (DODAB) concentration on main characteristics of PMMA/DODAB NPs synthesized by emulsion polymerization of MMA in the presence of DODAB (**d**–**f**). From dynamic light scattering (DLS), mean hydrodynamic diameter (Dz) (**a**,**d**), zeta—potential (ζ) (**b**,**e**) and polydispersity (P) (**c**,**f**) were determined for the dispersions in pure water. NPs in dispersion at 1.5 mM DODAB were synthesized over a range of [MMA]. NPs were also synthesized at 0.3 M MMA over a range of [DODAB].

The background for synthesizing PMMA/DODAB NPs has been the high miscibility of DODAB and PMMA due to weak but numerous intermolecular interactions [40]. The hybrid coatings using PMMA/DODAB materials joined the high biocompatibility of PMMA [21,50,51] with the antimicrobial activity of the quaternary ammonium moiety of DODAB [52,53,54,55]. Here we take advantage of PMMA biocompatibility and DODAB activity as immunoadjuvant to obtain hybrid NPs of PMMA/DODAB. One should also recall the high surface potential of DODAB nanostructures, bilayer fragments and bilayer vesicles [56] favoring the adsorption of oppositely charged antigens, an essential property for combining antigen and adjuvant. The main physical properties of PMMA/DODAB NPs obtained by emulsion polymerization of MMA in the presence of DODAB were on Figure 1.

Figure 1 showed the effect of MMA or DODAB concentration used during NPs synthesis on physical properties of PMMA/DODAB NPs. DODAB BF or DODAB dispersed as films on the bottom of the glass tube when used in the MMA polymerization medium yielded polymeric NPs further characterized by DLS for sizing, polydispersity (P) and zeta-potentials (ζ). Sizes (Dz) (Figure 1a), zeta-potentials (ζ) (Figure 1b) and polydispersities (P) (Figure 1c) were shown over a range of PMMA or DODAB concentrations. Nanometric size (50–80 nm) was mainly achieved using the DODAB BF during NPs synthesis over a range of MMA concentrations (Figure 1a). DODAB BF emulsified MMA better than DODAB films casted on the bottom of glass tubes. Therefore, in Figure 1a,d, NPs synthesized in the presence of DODAB BF (red points) were always smaller than those synthesized in the presence of DODAB films (blue points).

Over a range of low [MMA] (below 0.2 M MMA) in the reaction mixture, dispersions contained polydisperse NPs in contrast to the narrow size distribution obtained above 0.2 M MMA (Figure 1c). Thus [MMA] concentrations for nanometric sizes, high zeta-potential and narrow size distribution should be chosen above 0.2 M (concentration chosen was 0.3 M). Regarding the use of DODAB films for NPs synthesis, no signicant difference in zeta-potential and polydispersity was observed, but sizes tended to be always larger than the ones for NPs obtained from DODAB BF (Figure 1a–c).

At 0.3 M MMA, BFs confirmed their suitability to yield NPs sizes below 100 nm; DODAB films yielded always larger NPs (Figure 1d). This had already been observed on Figure 1a, where films yielded always larger NPs than those prepared in the presence of DODAB BF. Again, nanometric size (50–80 nm) was mainly achieved using the DODAB BF during NPs synthesis over a range of MMA concentrations (Figure 1d). DODAB BF emulsified MMA better than DODAB films casted on the bottom of glass tubes. Possibly, DODAB BF present in the aqueous reaction medium organized the growing chains of PMMA in the core of the bilayer fragments at early stages of the polymerization. The high miscibility between PMMA and DODAB led to hybrid NPs where DODAB could be localized both in the polymer matrix and on the NP surface. Curiously, DODAB affinity for PMMA has been shown to be higher than the one for cetyltrimethylammonium bromide (CTAB): whereas DODAB remains in the polymer matrix, CTAB diffuses through the polymer network reaching the outer water medium [19,40,57].

Figure 1e,f confirmed similar zeta-potentials and polydispersities for NPs synthesized in the presence of DODAB BF or DODAB films.

**Figure 2:**
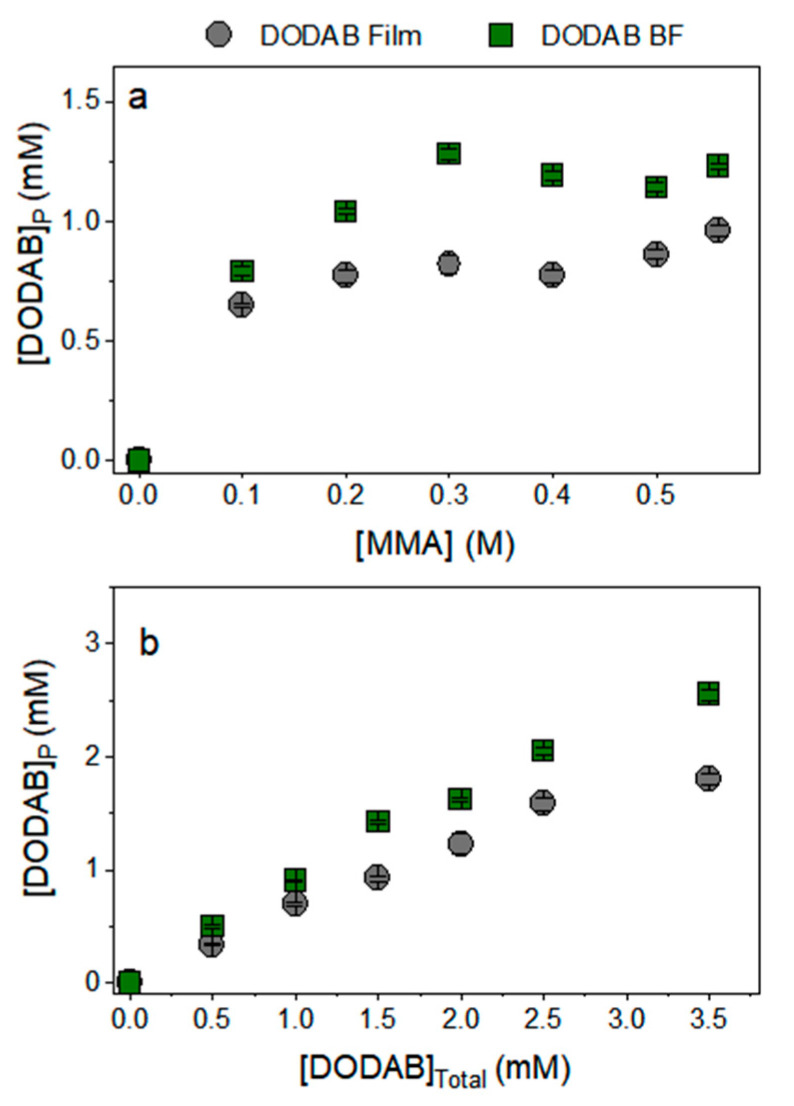
Incorporation of dioctadecyl dimethyl ammonium bromide (DODAB) in in poly(methyl methacrylate) (PMMA)/DODAB nanoparticles (NPs) as a function of methyl methacrylate concentration ([MMA]) using 1.5 mM DODAB (**a**) and as a function of DODAB concentration employing 0.3 M MMA (**b**).

Figure 2a showed the DODAB incorporation at 1.5 mM DODAB onto PMMA/DODAB NPs over a range of [MMA]. DODAB incorporation in the PMMA/DODAB NPs increased with [MMA] over a low range of [MMA] (0–0.3 M MMA). Above this range, further increasing [MMA] did not increase DODAB incorporation in the NPs.

From a certain MMA concentration it was not possible to disperse all PMMA as NPs due to lack of DODAB emulsifier; polymerization proceeded anyway yielding precipitated polymeric masses in the reaction mixtures; this occurred mainly over a range of [MMA] above 0.3 M (Figure 2a). Thus there is a need of establishing the saturation of PMMA with DODAB at a given concentration of MMA. For example, until 0.3 M MMA there was a linear dependence of DODAB incorporation from BF into PMMA/DODAB NPs (Figure 2a). However, it was observed that above 0.3 M MMA there were nonemulsified, precipitated polymer masses at the bottom of the assay tubes after NPs synthesis. For DODAB from films, the emulsification was even more hampered due to a lower availability of DODAB molecules to diffuse into the reaction mixture; at least for BF, the previous step of dispersing DODAB by sonication placed DODAB in a more suitable position as emulsifier for obtaining well dispersed polymeric NPs (Figure 2a,b). DODAB incorporation in NPs was always higher for polymerizations in the presence of BF than those in the presence of films (Figure 2). Furthermore, from DODAB added as BF, at 0.3 M MMA, there was a linear dependence of DODAB incorporation with the total DODAB concentration added to the reaction mixture, at least up to 1.5 mM DODAB (Figure 2b). In addition, all DODAB added could become incorporated in the NPs (Figure 2b). Above 1.5 mM DODAB added not all DODAB became part of the NPs meaning that the maximal DODAB incorporation had been achieved at a certain proportion of MMA/DODAB, namely 0.3 M MMA and 1.5 mM DODAB (Figure 2b).

The initial study determining the optimal molar ratio for MMA/DODAB during polymerization, namely 0.3:0.0015 MMA:DODAB, suggested this ratio for synthesizing the NPs to be evaluated as antigen presenters. The concentrations used for NPs synthesis in the next experiments were 0.3 M MMA and 0.0020 M DODAB, considering that the cationic DODAB usually adsorbs onto all glass [58] or plastic containers [33]. In other words, we synthesized the NPs under a slight excess of DODAB, even though complete removal of DODAB by centrifuging, discarding the supernatant and resuspending in pure water was routinely done. For the final NPs, [DODAB]_p_ was 1.62 mM.

**Figure 3:**
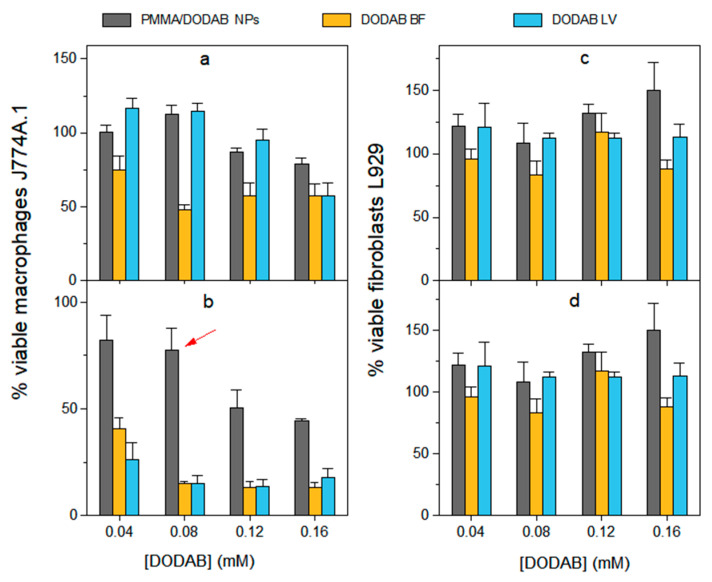
Cell viability of macrophages (J774A.1) or fibroblasts (L929) as affected by three different adjuvants containing dioctadecyl dimethyl ammonium bromide (DODAB) at 3 (**a**,**c**) and 24 h interaction time (**b**,**d**). The adjuvants were DODAB bilayer fragments (DODAB BF) (in yellow), DODAB bilayer vesicles (DODAB LV) (in blue) and poly (methyl methacrylate) (PMMA)/DODAB hybrid NPs (in gray). The DODAB concentration used for the three different adjuvants (PMMA/DODAB NPs, DODAB BF and DODAB LV) was indicated by the red arrow and corresponded to 0.08 mM DODAB.

In the presence of PMMA/DODAB NPs, DODAB BF and DODAB LV, determination of the viability of macrophages or fibroblasts in culture pointed out that DODAB concentration at 0.08 mM barely affected the macrophages and the fibroblasts after a period of 24 h interaction (Figure 3). In contrast with DODAB BF or DODAB LV, the PMMA/DODAB NPs resulted much less cytotoxic against the macrophages (Figure 3b). The [DODAB] selected to the next experiments on adjuvant activity of the NPs was selected as 0.08 mM DODAB.

One should notice that from a NPs dispersion of 20 mg/mL PMMA (determined from solid contents of the PMMA/DODAB dispersion) synthesized in the presence of 2.0 mM DODAB, after centrifuging to precipitate the NPs and determining the DODAB concentration in the supernatant as 0.38 mM the [DODAB]_p_ would be 1.62 mM. From this dispersion with 20 mg/mL PMMA and 1.62 mM DODAB, aliquots were taken to yield the four final [DODAB] on Figure 3, namely, 0.04, 0.08, 0.12 and 0.16 mM DODAB; for PMMA, final concentrations were 0.25, 0.50, 0.75 and 1.00 mg/mL, respectively. Curiously, there was no cytotoxicity against the fibroblasts in the presence of all three adjuvants over the 0–0.16 mM range of [DODAB] (Figure 3c,d). This result agrees with previous data on fibroblasts cell death in the presence of DODAB BF showing significant DODAB cytotoxicity only above 0.5 mM DODAB [59]. In summary, the use of DODAB mixed as a hybrid material with a biocompatible polymer such as PMMA advantageously reduced DODAB toxicity against the macrophages.

### 3.2. Ovalbumin Adsorption onto NPs, NPs/OVA Physical Properties and Colloidal Stability, and NPs/FITC-OVA Internalization in Macrophages

OVA adsorption onto PMMA/DODAB NPs was determined at 0.5 mg/mL PMMA over a range of added OVA concentrations. The reason for choosing this PMMA concentration was the possibility of comparing later on, the adjuvant effects of PMMA/DODAB with the previously described effects for PMMA/DODAB/PDDA NPs as adjuvants at this same concentration [21].

**Figure 4:**
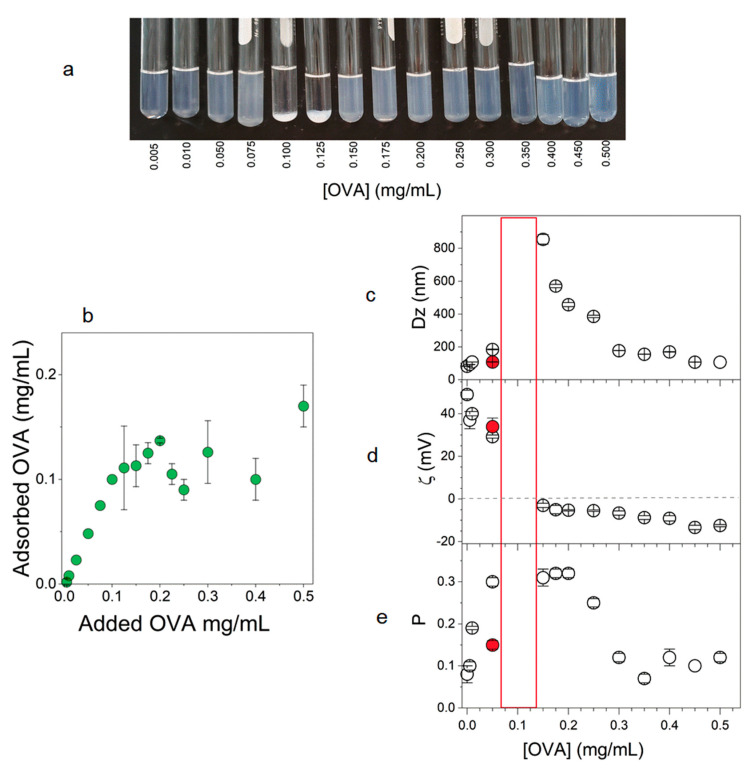
Poly(methyl methacrylate)/dioctadecyl dimethyl ammonium bromide (PMMA/DODAB) NPs at 0.5 mg/mL PMMA over a range of ovalbumin (OVA) concentrations: (**a**) photos of the dispersions; (**b**) OVA adsorption on NPs as a function of added OVA; (**c**) mean hydrodynamic diameter (Dz); (**d**) zeta-potential (ζ); (**e**) polydispersity of the dispersions (P). The stock solution of OVA used was 10 mg/mL whereas the red dot indicates properties of dispersions obtained from a 1 mg/mL OVA stock solution. The immunology tests were performed using the last stock solution of OVA. The red rectangle corresponds to the OVA concentration region of poor colloidal stability, meaning that DLS measurments could not be performed due to precipitation in the tubes.

The oppositely charged OVA adsorbed onto PMMA/DODAB NPs at 0.5 mg/mL PMMA over a range of [OVA] yielding the macroscopic features shown on Figure 4a. Upon charge neutralization and zeta-potentials around zero, there was precipitation and loss of colloidal stability around 0.100–0.125 mg/mL of OVA (Figure 4a,d).

This behavior is usually obtained for interaction between NPs and oppositely charged molecules such as silica/DODAB [37], and polystyrene sulfate NPs/DODAB [15,33]. Around neutralization, large aggregates precipitate and polydispersity increases substantially; basically, it is not possible to use the DLS technique due to the absence of colloids in dispersion. The determination of colloidal stability was important to define OVA concentration to be combined with 0.5 mg/mL PMMA. To obtain positively charged NPs, their concentrations should be smaller than 0.1 mg/mL; furthermore, at 0.1 mg/mL OVA, aggregation and precipitation hampered the use of this OVA concentration. The OVA concentration chosen was 0.05 mg/mL in combination with 0.5 mg/mL PMMA/DODAB NPs. The reason for choosing 0.5 mg/mL PMMA/DODAB NPs was to compare later on the adjuvant effect with the one induced by PMMA/DODAB/PDDA NPs described previously at this same concentration [21].

The red point on Figure 4c–e showing the physical properties of NPs/OVA were obtained from a 1 mg/mL OVA stock solution. As compared to NPs/OVA obtained from a 10 mg/mL OVA stock solution (black points), sizes and polydispersities were smaller for the NPs obtained from the 1 mg/mL OVA. This was observed previously for concentrated OVA stock solutions and attributed to OVA-OVA interactions leading to OVA aggregation in the stock solution that remains to a certain extent after further dilution [60]. Thus, the stock OVA solution used was the one at 1 mg/mL OVA (red points in Figure 4c–e).

The OVA adsorption onto PMMA/DODAB NPs at 0.5 mg/mL PMMA increased linearly with added OVA concentration up to 0.1 mg/mL of added OVA (Figure 4b). Above this concentration NPs became saturated with OVA and further adsorption was substantially reduced. Therefore, 0.05 mg/mL of added OVA was suitable to ensure that all OVA added was adsorbed onto the NPs, no free OVA remained in solution.

In comparison with PMMA/DODAB/PDDA NPs (zeta-potentials around 70 mV), the present system with PMMA/DODAB NPs exhibited smaller zeta-potentials (around 30 mV) but maximal OVA adsorption was similarly around 0.10–0.12 mg/mL for both systems [21]. This can be better understood from the compared sizes for both NPs. PMMA/DODAB/PDDA NPs (Dz = 217 nm) were larger than PMMA/DODAB NPs (Dz = 81 nm). At 0.5 mg/mL PMMA for both NPs, the total surface area available for OVA adsorption is larger for the smaller NPs explaining why the lower zeta-potential resulted in similar adsorption to the one obtained with the PMMA/DODAB/PDDA NPs with higher zeta-potential. Small sizes compensated for the lower zeta-potentials.

### 3.3. Immune Response of NPs/OVA In Vivo: Improved Production of IgG1 and IgG2a

**Figure 5:**
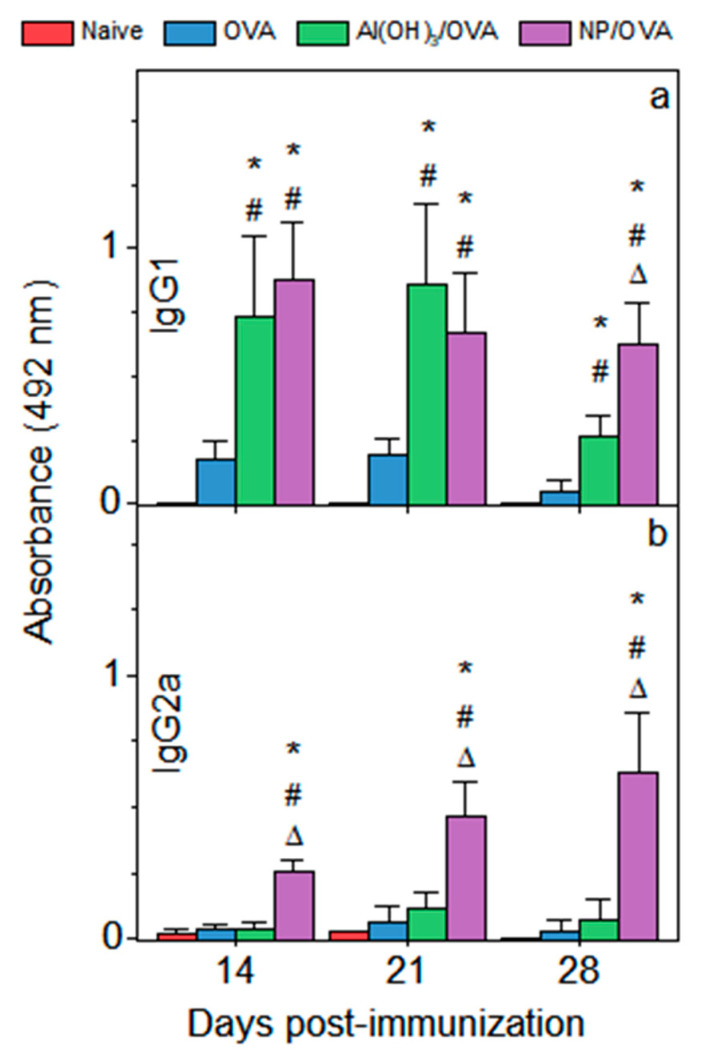
Antibody production in sera on days 14, 21 and 28 after immunization as induced by ovalbumin (OVA), Al(OH)_3_/OVA and NPs/OVA. The mean absorbance measured at 492 nm ± standard deviation was relative to the production of OVA-specific IgG1 (**a**) or IgG2a (**b**). Serum dilution to present IgG1 primary response was 1/1024 and IgG2a primary response was showed at 1/32. Secondary response of IgG1 was displayed at 1/512,000 whereas IgG2a secondary response was presented at 1/512. *p* < 0.05 compared to the naive group (#), *p* < 0.05 compared to the OVA group (*), *p* < 0.05 compared to the Al(OH)_3_/OVA group (∆).

IgG1 and IgG2a OVA-specific antibodies production was enhanced substantially using NPs/OVA for immunization (Figure 5). Furthermore, NPs were as efficient as Al(OH)_3_ as adjuvants inducing the IgG1 primary response on days 14 and 21 but surpassed alum regarding the secondary response on day 28 (Figure 5a). IgG2a production induced by NPs/OVA was substantially superior to the one induced by alum both for the primary and the secondary response, suggesting and important role of the novel NPs in promoting cellular immune response (Figure 5b). Similar NPs made of the biocompatible PMMA and a cationic polymer were previously used as adjuvants also yielding improved humoral response [21,43]. Emulsion polymerization of methyl methacrylate (MMA), producing poly(methyl methacrylate) (PMMA), is well described in the literature [61,62,63]. We have been studying the effect of adding cationic lipid, surfactant or polymer to obtain cationic PMMA NPs useful as immunoadjuvants. Adding a cationic polymer to obtain PMMA/cationic polymer NPs, the IgG1 production was about four times higher than the one induced by alum [21] whereas in the present work, adding the cationic lipid DODAB to obtain PMMA/cationic lipid NPs, the IgG1 production was similar to the one induced by alum (Figure 5a). Production of IgG2a induced by PMMA/cationic lipid (Figure 5b) was four times higher than the one induced by PMMA/cationic polymer [21]. The cationic polymer PDDA was shown to enhance humoral response at levels superior to those attributed to polyethyleneimine (PEI) or cetyltrimethylammonium bromide (CTAB) adsorbed to gold nanorods [64].

In order to obtain more quantitative data on IgG1 and IgG2a antibodies production, the endpoint titers were obtained in both cases and shown on Table 2. The qualitative data described on Figure 5 were reconfirmed by the titers’ determination. PMMA/DODAB NPs induced indeed a mixed Th1-Th2 response in contrast to OVA alone or alum as adjuvant. Although OVA alone induced a certain production of IgG1, the production of IgG2a was basically neglectable (Table 2).

In summary, the NP structure determines the magnitude of both humoral and cellular responses when used as adjuvants. The cationic component as the outermost layer imparts high surface potential to the NPs and is seen by the immune system as a foreign body. The hybrid cationic lipid/PMMA NPs exhibit a lower surface potential due to uniform distribution of the cationic lipid in the polymer matrix and are not seen as so foreigner by the immune system.

**Figure 6:**
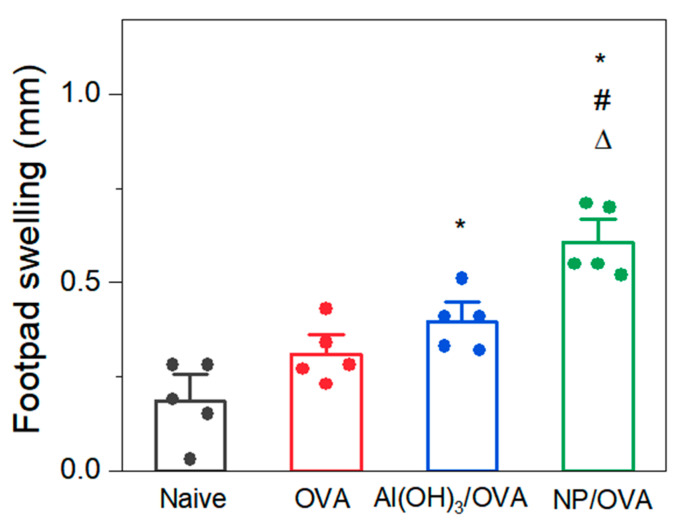
Delayed-type hypersensitivity reaction assessed as the mean footpad swelling ± standard deviation of animals immunized with ovalbumin (OVA), Al(OH)_3_/OVA or PMMA/DODAB/OVA (NP/OVA). *p* < 0.05 compared to the naive group (*), *p* < 0.05 compared to the OVA group (#), *p* < 0.05 compared to the Al(OH)_3_/OVA group (∆).

Figure 6 shows the footpad swelling (FS) in mm for PMMA/DODAB NPs as compared to alum. The FS was 0.7 mm (Figure 6) and lower than the one previously determined for DODAB BF/OVA by Rozenfeld and coworkers, which was 1.3 mm [11]. Whereas the zeta-potential for PMMA/DODAB/OVA in this work is about 34 mV (Figure 4d), the zeta-potential for DODAB BF/OVA was determined as 21 mV [11]. The less charged nanostructure apparently induced the larger cellular response.

**Figure 7:**
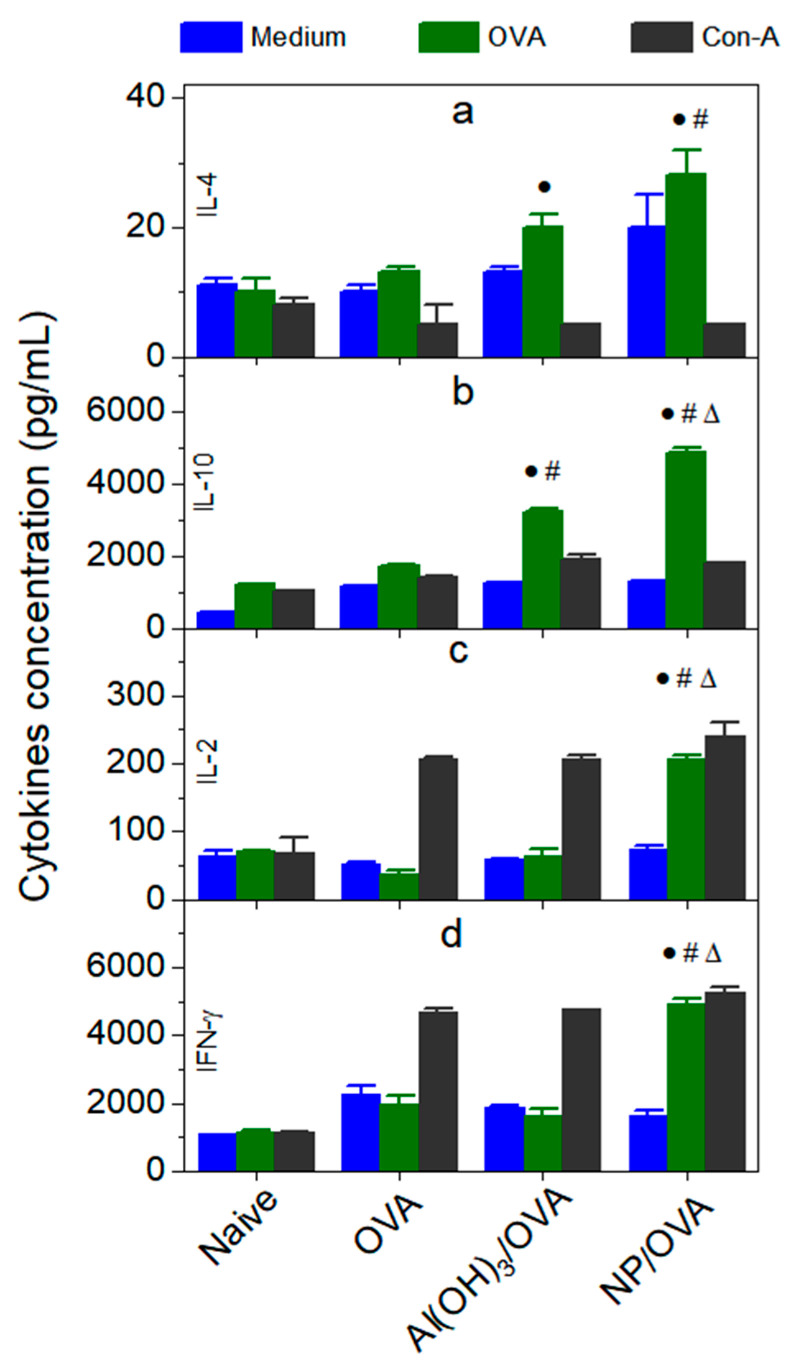
Production IL-4 (**a**), IL-10 (**b**), IL-2 (**c**) and IFN-γ (**d**) (pg/mL) elicited by cultured spleen cells obtained from naïve or animals immunized with ovalbumin (OVA), Al(OH)_3_/OVA, PMMA/DODAB/OVA (NPs/OVA). *p* < 0.05 compared to the naive group (•), *p* < 0.05 compared to the OVA group (#), *p* < 0.05 compared to the Al(OH)_3_/OVA group (∆). Splenocytes were challenged with OVA, medium (negative control) and concanavalin-A (Con-A) (positive control).

Some cytokines elicited by medium (negative control), OVA or concanavalin-A in cultured spleen cells of animals previously immunized with water (naive), OVA, Al(OH)_3_/OVA or PMMA/DODAB/OVA NPs allowed to reconfirm the intensity of humoral and cellular immune responses (Figure 7). Whereas IL-4 enhancement means improved humoral response (Figure 7a) and IL-10 enhancement relates to both humoral and cellular responses (Figure 7b), IL-2 (Figure 7c) and IFN-γ are typically produced in enhanced cellular immune response (Figure 7d). This profile perfectly agrees with previous results reported in this work where significant enhancements in both cellular (Figure 5b and Figure 6) and humoral immune responses were indeed described (Figure 5a). IL-10 enhancement means a regulatory response against the pro-inflammatory effect of NPs/OVA in mice.

In the presence of DODAB and poly(diallyl dimethylammonium chloride) (PDDA), emulsion polymerization of MMA yielded PMMA/DODAB/PDDA NPs [21,43,45]. These hybrid, PMMA-based NPs predominantly contained PMMA, a biocompatible polymer, displaying absence of cytotoxicity when tiny amounts of cationic components were used, as shown in this work. The comparison between PMMA/DODAB NPs (this work) and PMMA/DODAB/PDDA [21] showed some differences in the type of immune response. The former NPs induced preferentially a Th-1 response (higher IgG2a, IFN-γ and lower IL-10 for down regulation of inflammatory response induced by the PMMA/DODAB NP) as compared to the PMMA/DODAB/PDDA NPs that generated higher Th-2 response (lower IgG2a, IFN-γ and higher IL-10 attempting to reduce the NPs induced inflammatory effect).

The comparison between PMMA/DODAB NPs and DODAB bilayer fragments (BF) regarding the DTH response measured as footpad swelling (FS) also showed the mild inflammatory effect of the PMMA/DODAB NPs (FS = 0.70 mm) in comparison to DODAB BF (FS = 1.3).

**Figure 8:**
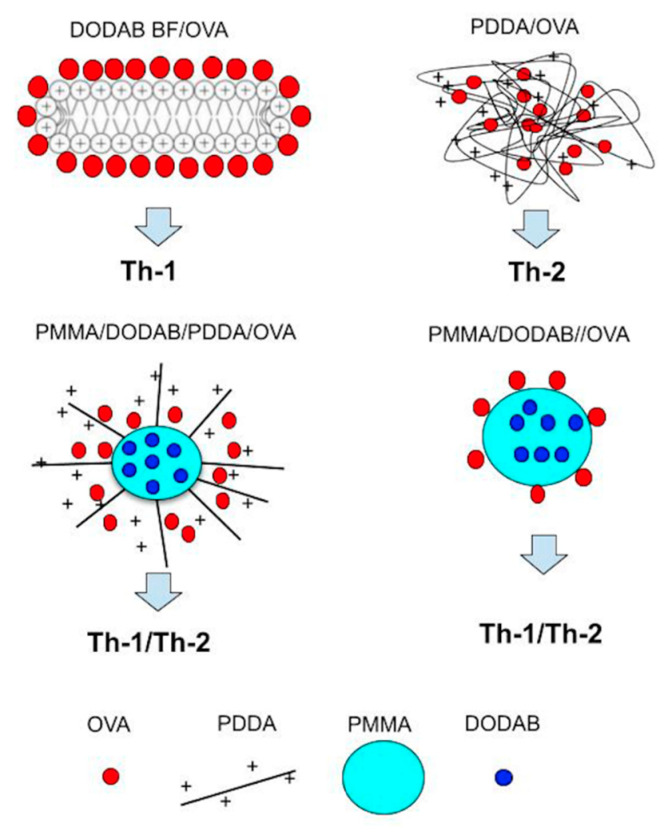
Cationic nanostructures from biocompatible polymer and cationic lipid and/or cationic polymer.

Figure 8 shows schematically four different cationic nanostructures and their immune responses as studied in our group over the last 25 years.

## 4. Conclusions

PMMA/DODAB NPs with nanometric sizes, high zeta-potential and narrow size distribution were obtained by emulsion polymerization of MMA in the presence of DODAB (at 0.3 M MMA and 2.0 mM DODAB). From DODAB films for NPs synthesis sizes were always larger than the ones for NPs obtained from DODAB BF. PMMA/DODAB NPs exhibited functional behavior as immunoadjuvants. They were non cytotoxic against macrophages and fibroblasts in culture, combined well with OVA, as model antigen, were nanometric, showing high zeta-potential, low polydispersity and high colloidal stability in water dispersions. NPs/OVA induced high production of OVA-specific IgG1 and IgG2a in immunized mice, high delayed type hypersensitivity reaction, high production of IFN-γ, regulatory cytokine IL-10, IL-2 and IL-4. Quantitative determination of IgG1 and IgG2a titers reconfirmed the induction of a mixed Th1-Th2 response induced by the PMMA/DODAB/OVA NPs. In summary, there was enhanced humoral and cellular responses against OVA carried by the novel biocompatible adjuvant.

## Figures and Tables

**Table 1:** Solutions or dispersions in pure water used in mice immunization.

Group	Solution/ Dispersion	OVA mg/mL	Al(OH)_3_ mg/mL	PMMA mg/mL	DODAB mM
1	Ultrapure water	0	0	0	0
2	Ovalbumin (OVA)	0.05	0	0	0
3	Al(OH)_3_/OVA	0.05	0.10	0	0
4	PMMA/DODAB/OVA	0.05	0	0.50	0.08 ^1^

^1^ 0.08 mM DODAB is equivalent to 0.05 mg/mL.

**Table 2:** Endpoint titer of OVA-specific IgG1, IgG2a and IgG1/IgG2a ratio determined 21 days after immunization.

Group	IgG1	IgG2a	IgG1/IgG2a
OVA	1434 ± 360	14 ± 10	102
Al(OH)_3_/OVA	35,200 ± 13,000	58 ± 14	607
NPs/OVA	41,600 ± 15,648	358 ± 132	116

## Data Availability

All data available are reported in the article.
